# Tick-borne Encephalitis Virus, Zealand, Denmark, 2011

**DOI:** 10.3201/eid1907.130092

**Published:** 2013-07

**Authors:** Anders Fomsgaard, Mette E. Fertner, Sandra Essbauer, Alex Y. Nielsen, Stefan Frey, Pontus Lindblom, Per-Eric Lindgren, Rene Bødker, Manfred Weidmann, Gerhard Dobler

**Affiliations:** University of Southern Denmark, Odense, Denmark (A. Fomsgaard);; Statens Serum Institut, Copenhagen, Denmark (A. Fomsgaard, M.E. Fertner, A.Y. Nielsen);; Institut für Mikrobiologie der Bundeswehr, Munich, Germany (S. Essbauer, S. Frey, G. Dobler);; Linköping University, Linköping, Sweden (P. Lindblom, P.-E. Lindgren);; Technical University of Denmark, Copenhagen (R. Bødker);; Abteilung Virologie, Universitätsmedizin Göttingen, Göttingen, Germany (M. Weidmann)

**Keywords:** Tick-borne, encephalitis, Denmark, flavivirus, Ixodes ricinus, emerging disease, vector-borne infections, ticks, ticks, viruses

**To the Editor:** In Scandinavia, the incidence of tick-borne encephalitis (TBE) is increasing and expanding its geographic range (*1*). TBE virus (TBEV) types TBEV-Eur and TBEV-Sib occur in Estonia and Finland, along with 2 tick species, *Ixodes persulcatus* and *I. ricinus*. In Denmark, TBE has been reported since the 1950s only from the isolated Bornholm Island in the Baltic Sea with an incidence of ≈4 cases per 100,000 persons (*2*). Statistical climate-matching models based on the known spatial distribution of TBEV indicate that the present North Zealand climate also would support TBEV-Eur transmission cycles (*3*). Recently (2008 and 2009), we reported TBE in 2 persons who had histories of tick-bite and originated from a single location in a small forest area (Tokkekøb Hegn) in North Zealand where TBE was previously unrecognized (*4*).

To determine whether TBE was established in this possibly new TBE focus in mainland Denmark outside Bornholm, we collected ticks by flagging (*4*) from 3 sites at Tokkekøb during June–July 2011. The 3 sites yielded 896 ticks (854 nymphs, 22 male adults, 20 female adults) in 24 pools. A fourth site at Grib Forest 10 km to the north yielded 198 ticks (183 nymphs, 9 male adult, 6 female adults) in 13 pools. Flagging was repeated in September 2011 at Tokkekøb to confirm the presence of TBEV and to obtain material suitable for virus isolation. Here, we obtained 7 pools (100 nymphs each) and 1 pool with adults (15 male, 15 female). In September 2011, we also obtained 13 pools (738 nymphs, 37 male adults, 41 female adults) at 3 suspected TBE locations on Bornholm Island. In addition, 1,073 ticks in 58 pools were collected in 2010 and 2011 from deer inspected by the National Center for Wildlife Health from 54 various locations (Figure, panel A). All ticks were identified as *I. ricinus* on the basis of morphology. For TBEV-specific real-time PCR (*5*), ticks were homogenized in 0.5 mL nucleic acid extraction buffer and RNA/DNA extracted from 0.2 mL homogenate by using the MagNA Pure total NA kit (Roche, Indianapolis, IN, USA). Three of 37 pools (2 with nymphs, 1 with adult females) from Tokkekøb were TBEV RNA positive. None of 58 tick pools from other locations in Denmark or Bornholm were positive for TBEV but contained other pathogens (*6*). Five of the 8 pools obtained from the second flagging session (all nymphs) in Tokkekøb were TBEV PCR positive, and 2 yielded isolates (T2, T3) in VeroB4 cell culture. Considering that the duration of the nymphal stage in *I. ricinus* is usually only 1 or 2 years in northern Europe (*7*), the repeated identification of TBEV in nymphs at the same location in 2009 and 2011 indicates establishment of a new focus of endemic TBEV in Denmark.

Phylogenetic analysis of TBEV-E sequences (1,488 nt) of central European (*8*) and Scandinavian TBEV strains did not group the Zealand isolate T2 (T3 was not sequenced) with the Bornholm strain but into a subclade with 2 isolates from Sweden, Torö-2003 (*9*) (GenBank accession no. DQ401140) and Saringe-2009 (GenBank accession no. KC469073); an isolate from Norway (GenBank accession no. EF565947), and isolates from North Bohemia (Czech Republic). The Bornholm strain located into a different subclade containing various sequences from South and Central Bohemia (Figure, panel B). TBEV sequences from the Baltics and Finland locate to a spate clade. The missing link between the isolates from Bornholm and Zealand also was observed in a median joining network analysis (Splits Tree program, Epsilon1 [www.splitstree.org], 2,000 iterations [data not shown]).

**Figure:**
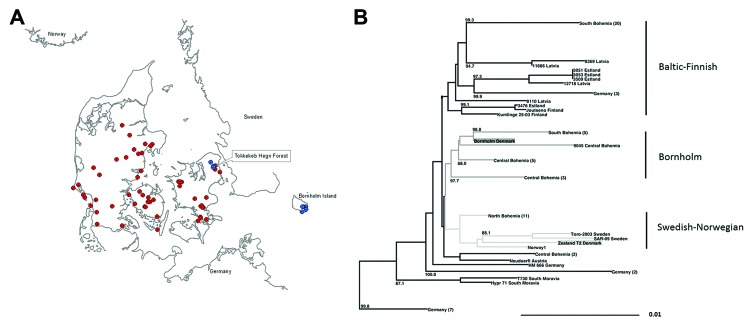
A) Tick collection areas in Denmark. Red indicates ticks sampled from animals; blue indicates flagging. B) Neighbor-joining phylogenetic analysis of a 1,488-nt set of 78 tick-borne encephalitis virus (TBEV)–Eur E gene sequences including reference strains Neudoerfl (Austria) and Hypr 71 (South Moravia) performed in ClustalW with a 1,000 bootstrap approach (LASERGENE, MEGALIGN, DENDROSCOPE) outgrouped to Louping ill virus (data not shown). Sequence designations of central European strains as in (*8*). Dark gray indicates Denmark Bornholm clade; light gray indicates Swedish-Norwegian clade. For simplicity, some subtrees were collapsed; these are designated with region and number of sequences in the collapsed subtree in brackets.

Two severe clinical cases of TBE connected to this new focus occurred in 2008–2009 (*4*). To search for additional missed clinical TBE cases from this area, we examined serum and cerbrospinal fluid of 96 patients (2007–2009) in whom encephalitis developed after tick bite; these samples were found negative for *Borrelia* spp. by antibody ELISA and PCR (Technical Appendix). To assess anti-TBEV seroprevalence, we also tested serum from 78 patients experiencing “summer flu” who had histories of tick bite; this serum was submitted by general practitioners in North Zealand during July–November 2010 (Technical Appendix). Except for 1 patient infected in Bornholm and 2 patients infected in Sweden, none were positive by ELISA (Enzygnost Anti-TBE/FSME Virus [IgG, IgM] Siemens, Erlangen, Germany) or PCR (Technical Appendix). Since the 1980s, Sweden has experienced a 4-fold increase in human TBE incidence, with spread southwest (*10*). The emergence of the TBEV strain T2 closely related to isolates from Sweden may be a continuation of this geographic trend. A previous antibody study found 3 deer positive for TBEV in Zealand-Falster (*2*); however, without convincing neutralization data, this finding is not confirmed. The lack of TBEV viremia and seropositivity among the patients in Zealand who had histories of tick bites supports a recent introduction to the new focus. Thus, 2 distinct introductions of TBEV have occurred in Denmark. The underlining environmental or climatic factors driving this geographic trend remain unknown.

Technical Appendix Overview of serum samples collected and tested for tick-borne encephalitis virus and antibodies in Denmark outside Bornholm, 2011.
