# Supplementary material for: Tick-borne Encephalitis Virus, Zealand, Denmark, 2011
Source: Emerg Infect Dis. 2013 Jul;19(7):1171–3. doi: 10.3201/eid1907.130092 (PMC3903456; doi:10.3201/eid1907.130092)
Supplement: Technical Appendix — Overview of serum samples collected and tested for tick-borne encephalitis virus and antibodies in Denmark outside Bornholm, 2011. [file 13-0092-Techapp-s1.pdf]

Article DOI: http://dx.doi.org/10.3201/eid1907.130092

Tick-borne Encephalitis Virus, Zealand, Denmark, 2011

Technical Appendix

Technical Appendix Table. Overview of serum samples collected and tested for tick-borne encephalitis virus and antibodies in Denmark outside Bornholm, 2011

Variable	*Borrelia* samples	Summer flu samples
No. samples	96	79
No. patients	96	78
Sex	55 F, 41 M	46 M, 32 F
Age, y (mean)	3–87 (41)	4-84 (49)
Period	2007 Jul 3–2009 Aug 14	2010 Jul 21–2010 Nov 4
Results	2 IgG positive and 1 IgG and IgM positive	None positive in PCR or serology
